# The Effects of Exposure to Mephedrone During Adolescence on Brain Neurotransmission and Neurotoxicity in Adult Rats

**DOI:** 10.1007/s12640-018-9908-0

**Published:** 2018-04-30

**Authors:** Katarzyna Kamińska, Karolina Noworyta-Sokołowska, Anna Górska, Joanna Rzemieniec, Agnieszka Wnuk, Adam Wojtas, Grzegorz Kreiner, Małgorzata Kajta, Krystyna Gołembiowska

**Affiliations:** 1 Department of Pharmacology, Institute of Pharmacology, Polish Academy of Sciences, 12 Smętna, 31-343 Kraków, Poland; 2 Department of Experimental Neuroendocrinology, Institute of Pharmacology, Polish Academy of Sciences, 12 Smętna, 31-343 Kraków, Poland; 3 Department of Brain Biochemistry, Institute of Pharmacology, Polish Academy of Sciences, 12 Smętna, 31-343 Kraków, Poland

**Keywords:** Mephedrone, Adolescence, Neurotransmitters, Microdialysis, Neurotoxicity

## Abstract

According to the European Drug Report (2016), the use of synthetic cathinones, such as mephedrone, among young people has rapidly increased in the last years. Studies in humans indicate that psychostimulant drug use in adolescence increases risk of drug abuse in adulthood. Mephedrone by its interaction with transporters for dopamine (DAT) and serotonin (SERT) stimulates their release to the synaptic cleft. In animal studies, high repeated doses of mephedrone given to adolescent but not adult mice or rats induced toxic changes in 5-hydroxytryptamine (5-HT) neurons. The aim of our study was to investigate the effects of mephedrone given in adolescence on brain neurotransmission and possible neuronal injury in adult rats. Adolescent male rats were given mephedrone (5 mg/kg) for 8 days. In vivo microdialysis in adult rats showed an increase in dopamine (DA), 5-HT, and glutamate release in the nucleus accumbens and frontal cortex but not in the striatum in response to challenge dose in animals pretreated with mephedrone in adolescence. The 5-HT and 5-hydroxyindoleacetic acid contents decreased in the striatum and nucleus accumbens while DA turnover rates were decreased in the striatum and nucleus accumbens. The oxidative damage of DNA assessed with the alkaline comet assay was found in the cortex of adult rats. Therefore, the administration of repeated low doses of mephedrone during adolescence does not seem to induce injury to 5-HT and DA neurons. The oxidative stress seems to be responsible for possible damage of cortical cell bodies which causes maladaptive changes in serotonergic and dopaminergic neurons.

**Electronic supplementary material:**

The online version of this article (10.1007/s12640-018-9908-0) contains supplementary material, which is available to authorized users.

## Introduction

New substances, in particular stimulants, such as mephedrone, alpha-PVP, MDPV, and pentedrone, have been associated with a range of serious harms in Europe including acute poisonings and deaths (EMCDDA 2016; Eur Drug Rep 2016). In 2014, synthetic cathinones accounted for more than 15% of all seizures of new psychoactive substances (NPSs). Estimated 17.8 million young adults in Europe used psychoactive drugs in the last year. In the most recent survey (2014/2015), last year use of mephedrone among young people aged 16 to 24 was estimated at 1.9% (Eur Drug Rep 2016).

Mephedrone (4-methylmethcathinone) is a synthetic derivative of cathinone, an ingredient found in khat (*Catha edulis*, Forsk), a shrub, the leaves of which are chewed as a recreational drug in Africa and the Arabian Peninsula (Feyissa and Kelly 2008). Mephedrone, a β-ketoamphetamine with structural analogy to substituted amphetamines, is a powerful psychostimulant similar to methamphetamine and entactogen 3,4-methylenedioxymethamphetamine (MDMA; ecstasy) (Schifano et al. 2011). The study of Aarde et al. (2013) provides evidence of stimulant and abuse liability of mephedrone in rats. Like amphetamines, mephedrone causes locomotor activation in rats (Motbey et al. 2011) and other psychomimetic effects, such as euphoria, elevated mood, and sexual stimulation (Kehr et al. 2011). Mephedrone interacts with plasma membrane transporters for dopamine (DAT) and 5-hydroxytryptamine (5-HT, SERT), blocks the neurotransmitter reuptake (Baumann et al. 2012; Hadlock et al. 2011; Simmler et al. 2014), and stimulates their release to the synaptic cleft (Kehr et al. 2011). We (Gołembiowska et al. 2016) and others (Baumann et al. 2012; Kehr et al. 2011) have shown that mephedrone stimulates dopamine (DA) and 5-HT release in vivo in rats. The issue of whether mephedrone causes neurotoxicity, like methamphetamine and MDMA, remains controversial. PET imaging studies in methcathinone users revealed reduced DAT density, suggesting a loss of DA terminals (McCann et al. 1998). However, in animal studies, mephedrone alone given in a binge-like regimen (4 × 40 mg/kg every 2 h) was not toxic for DA nerve endings in the mouse striatum, but it significantly enhanced the neurotoxic effects of methamphetamine and MDMA (Angoa-Pérez et al. 2013; Anneken et al. 2015). Mephedrone administered in the same way also did not cause toxicity to 5-HT nerve endings of the mouse hippocampus and did not influence methamphetamine and MDMA toxic effects (Angoa-Pérez et al. 2014). Moreover, den Hollander et al. (2013) demonstrated that mephedrone exposure twice daily for 4 days at a dose of 30 mg/kg to rats and mice produced no significant changes in brain monoamine levels. Conversely, Hadlock et al. (2011) reported a rapid decrease in DA and 5-HT transporter function after four doses of 10 and 25 mg/kg to rats at an ambient temperature of 27 °C. Motbey et al. (2012) failed to find changes in DA and 5-HT tissue levels 7 weeks after a 10-day treatment with 30 mg/kg of mephedrone in spite of acute increases in 5-HT and reductions in DA metabolism in adolescent rats that had been exposed to mephedrone. However, the impairment of recognition memory observed 1 month after cessation of treatment suggests possible neurotoxic and neuropsychiatric effects of mephedrone. Martinez-Clemente et al. (2014) and López-Arnau et al. (2015) evidenced mephedrone neurotoxicity in adolescent mice and rats by using a dosing schedule which better matched mephedrone pharmacokinetics and by exploring brain areas other than the striatum. A multiple-dose-per-day administration schedule (3 × 25 mg/kg/day in adolescent rats or mice) which mimicked the widespread use of mephedrone in dance clubs induced a DA and 5-HT transporter loss accompanied by a decrease in tyrosine hydroxylase and tryptophan hydroxylase 2 in the frontal cortex and hippocampus but not in the striatum of mice and rats (Martinez-Clemente et al. 2014; López-Arnau et al. 2015). The decrease in transporter level and enzyme markers points to an injury of the nerve endings (Escubedo et al. 2005). It is postulated that mephedrone exerts its neurotoxic actions through oxidative stress which damages components of the cell membrane, nucleus, and mitochondria, leading to complete cell degradation (Ciudad-Roberts et al. 2016).

Recreational use of psychostimulants during adolescence has been associated with alterations in brain structure and function (Squeglia et al. 2009). As shown in human studies, psychostimulant drug use during adolescence increases risk of drug abuse in adulthood (Izenwasser 2005). The changes occurring in neurotransmitter systems during childhood/young adulthood could affect subject’s response in a way that is different from a normal response in adults (Izenwasser 2005). For instance, in animals, the levels of DA and other markers of transmitter activity in the striatum increase until puberty (Noisin and Thomas 1988). It is important to examine psychoactive drug effects in the adolescent population since there may be an increased vulnerability to the effects of drugs during this period. Specifically, transformations in the prefrontal cortical regions and limbic pathways may contribute to increased novelty-seeking behaviors (Spear 2000). Furthermore, it has to be considered whether psychoactive drug use in adolescence has impact on drug response in adulthood. So far, this issue has not been explored.

The aim of this study was to investigate the effects of mephedrone administered to rats during adolescence in a pattern that mimicked taking multiple doses over time to maintain the drug effect. We studied the effect of mephedrone on DA, 5-HT, and glutamate extracellular level in the frontal cortex, striatum, and nucleus accumbens after animals reached adulthood. Due to similarity of mephedrone to amphetamines, we investigated the risk of oxidative stress by measuring oxidative DNA damage. Changes in monoamine levels were also assessed in order to assess neuronal injury.

## Materials and Methods

### Animals

The study was carried out on male *Wistar-Han* rats (Charles Rivers, Sulzfeld, Germany) weighing 90–100 g. The animals arrived to our facility on the 21st day of age (postnatal day, PND) and were allowed to acclimate; then, they were randomly assigned to control and drug-treated groups. The animals were housed in temperature- and humidity-controlled rooms on a 12-h light/12-h dark cycle and had free access to tap water and standard laboratory food. The experiments were conducted in strict accordance with European legal regulations concerning experiments on animals (Directive 2010/63/EU for animal experiments). The experimental protocols were approved by the Local Ethics Commission for Experimentation on Animals (permit number: 1274/2015).

### Drugs and Reagents

Mephedrone was purchased from Toronto Research Chemicals, Inc. (Canada). The chemicals used for high-performance liquid chromatography (HPLC) were obtained from Merck (Warsaw, Poland), while ketamine hydrochloride and xylazine hydrochloride came from Biowet (Puławy, Poland). The chemicals used for comet assay were purchased from Trevigen (Gaithersburg, MD). Sucrose was from Merck (Warsaw, Poland), while Triton from SERVA Electrophoresis (Heidelberg, Germany).

### Treatment

**Scheme 1:**
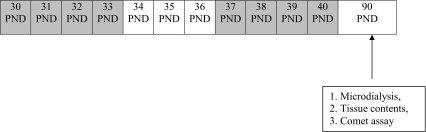
A schematic presentation of chronic mephedrone (8 × 5 mg/kg) administration during adolescence. Gray bars indicate days of administration; PND postnatal day

Administration of mephedrone started when rats attained PND 30. Rats were injected with mephedrone at room temperature in a dose of 5 mg/kg for 4 days from 30 to 33 PNDs which represent an early adolescence period (Cox et al. 2014), and after a 3-day break, another 4-day administration started from 37 to 40 PNDs representing a middle adolescence period (Cox et al. 2014). The pattern of drug injections is displayed in Scheme 1. Microdialysis experiments (1), determination of tissue contents (2), and comet assays (3) were conducted on separate groups of animals. All biochemical experiments were performed when rats reached adulthood at 90 PNDs. Mephedrone was dissolved in 0.9% NaCl and was administered intraperitoneally (ip). The control groups received the corresponding volume of 0.9% NaCl according to the same administration schedule as in the mephedrone-treated animals.

### Brain Microdialysis

Animals were anesthetized with ketamine (75 mg/kg) and xylazine (10 mg/kg), and vertical microdialysis probes MAB 4.15.4.Cu, MAB 4.15.2.Cu, and MAB 4.15.3.Cu (AgnTho’s, Sweden) were implanted into the striatum, nucleus accumbens, and frontal cortex using the following coordinates: AP + 1.8, L ± 3.0, V − 7.0; AP + 1.6, L ± 1.1, V − 8.0; and AP + 2.8, L ± 0.8, V − 6.0 from the dura, respectively (Paxinos and Watson 1998). On the next day, probe inlets were connected to a syringe pump (BAS, IN, USA) which delivered artificial cerebrospinal fluid (aCSF) composed of the following [mM]: NaCl 147, KCl 2.7, MgCl_2_ 1.0, and CaCl_2_ 1.2 (pH 7.4) at a flow rate of 2 μl/min. After 2 h of the washout period, three basal dialysate samples were collected every 20 min; then, animals were injected subcutaneously with mephedrone as indicated in figure captions and fraction collection continued for 180 min, respectively. At the end of the experiment, the rats were sacrificed and their brains were histologically examined and verified for probe placement.

#### The Measurement of Extracellular Concentration of DA, 5-HT, and Glutamate

The DA and 5-HT concentrations in dialysate fractions were analyzed by HPLC with coulochemical detection. Chromatography was performed using an UltiMate 3000 system (Dionex, USA), Coulochem III coulochemical detector (model 5300, ESA, USA) with 5020 guard cell, 5014B microdialysis cell, and Hypersil Gold C18 analytical column (3 μm, 3 × 100 mm; Thermo Scientific, USA). The mobile phase was composed of 0.1 M potassium phosphate buffer adjusted to pH 3.6, 0.5 mM Na_2_EDTA, 16 mg/l 1-octanesulfonic acid sodium salt, and 2% methanol. The flow rate during analysis was set at 0.7 ml/min. The applied potential of a guard cell was 600 mV, while those of microdialysis cells were E1 = − 50 mV and E2 = 300 mV with a sensitivity set at 50 nA/V. The chromatographic data were processed by Chromeleon v. 6.80 (Dionex, USA) software run on a personal computer.

Glutamate in extracellular fluid was measured by HPLC with electrochemical detection after derivatization with OPA/sulfite reagent to form isoindole-sulfonate derivative. Chromatography was performed using an UltiMate 3000 pump (Dionex, USA), an LC-4B amperometric detector with a cross-flow detector cell (BAS, IN, USA), and a HR-80 column (80 × 4.6 mm, 3 μm; ESA, Inc., USA). The mobile phase consisted of 100 mM monosodium orthophosphate at pH 4.6 and 4% methanol. The flow rate was 0.9 ml/min, and the applied potential of a 3-mm glassy carbon electrode was set at + 600 mV at a sensitivity of 5 nA/V. Glutamate derivative peak was compared with the respective standard, and the data were processed using Chromax 2005 (Pol-Lab, Warszawa, Poland) software on a personal computer.

### The Measurement of the Tissue Content of DA, 5-HT, and Their Metabolites

Animals were sacrificed by decapitation at 90 PNDs. Brains were removed, and several brain regions including the frontal cortex, striatum, and nucleus accumbens were dissected in anatomical borders. The tissue levels of DA, 5-HT, 3,4-dihydroxyphenylacetic acid (DOPAC), homovanillic acid (HVA), and 5-hydroxyindoleacetic acid (5-HIAA) were measured using a HPLC system with electrochemical detection. Tissue samples of brain structures were homogenized in ice-cold 0.1 M HClO_4_ and were centrifuged at 10,000×*g* for 10 min at 4 °C. The supernatant (3–5 μl) was injected into a HPLC system. The chromatographic system consisted of an LC-4C amperometric detector with a cross-flow detector cell (BAS, IN, USA), an UltiMate 3000 pump (Thermo Scientific, USA) and a HR-80 column (80 × 4.6 mm, 3 μm; ESA, Inc., USA). The mobile phase consisted of 0.1 M KH_2_PO_4_, 0.5 mM Na_2_EDTA, 80 mg/l sodium 1-octanesulfonate, and 4% methanol, adjusted to pH 3.7 with 85% H_3_PO_4_. The flow rate was 1 ml/min. The potential of a 3-mm glassy carbon electrode was set at 700 mV with sensitivity of 5 nA/V. The temperature of the column was maintained at 30 °C. The Chromax 2007 program (Pol-Lab, Warszawa, Poland) was used for data collection and analysis.

### Comet Assay

#### Preparation of Nuclear Suspension

Animals were killed 3 or 60 days after termination of drug treatments. The whole cortex was separated in anatomical borders. Next, the brain tissue was minced with a surgical scalpel and homogenized in a manual homogenizer with homogenizing solution containing 0.25% Triton. The homogenate was filtered and centrifuged at 850×*g* for 10 min. Thereafter, the supernatant was discarded, while the pellet was resuspended in the same volume of homogenization medium without Triton and centrifuged for 10 min at 850×*g*. The sediment was washed once more in the same way and centrifuged at 600×*g* for 8 min. The pellet was resuspended in 0.8 ml of homogenization solution without Triton, mixed with 4.2 ml of purification medium, and centrifuged at 19,000×*g* for 45 min. The nuclei were obtained as a transparent sediment at the bottom. The pellet was resuspended in 0.5 ml of 2.0 M sucrose and was layered over a sucrose gradient (2.6, 2.4 M bottom to top). The gradient was allowed to stand for 3 h at 0 °C before use. Fractionation of the nuclei was achieved by centrifugation at 19,000×*g* for 45 min.

#### Alkaline Comet Assay

The nuclei were added to a tube with 200 μl of PBS (without Ca^2+^ and Mg^2+^) and mixed gently. The suspension was mixed with LMA agarose and transferred immediately onto comet slides. The slides were placed at 4 °C in the dark for 10 min. Then, the slides were immersed in prechilled lysis solution and left at 4 °C in the dark for 30 min. The buffer was drained, and the slides were immersed in alkaline unwinding solution and left for 45 min in the dark. In the next step, electrophoresis was run at 21 V for 30 min. After electrophoresis, the slides were washed first with H_2_O and next with 70% ethanol and dried at 45 °C for 10 min. The slides were then covered with a dye and allowed to dry completely at room temperature in the dark. On the next day, the slides were examined under a fluorescent microscope. DNA damage was presented as an olive tail moment. Olive tail moment is defined as the product of the tail length and the fraction of total DNA in the tail. Tail moment incorporates a measure of both the smallest detectable size of migrating DNA (reflected in the comet tail length) and the number of damaged pieces (represented by the intensity of DNA in the tail). The olive tail moment is calculated according to the following formula: Olive tail moment = (Tail mean − Head mean) × Tail %DNA / 100.

### Data Analysis

Repeated measures ANOVA followed by Tukey’s post hoc test were performed to analyze drug effect on DA, 5-HT, and glutamate release in the rat brain regions. All obtained data were presented as a percent of the basal level assumed to be 100%. DNA damage in comet assay and tissue content of DA, 5-HT, and their metabolites were tested using one-way ANOVA followed by Tukey’s multiple comparison test.

## Results

### The Effect of Repeated Administration of Mephedrone During Adolescence on Extracellular Level of DA, 5-HT, and Glutamate Measured in Adulthood (90 PNDs) in the Rat Striatum, Nucleus Accumbens, and Frontal Cortex

#### Striatum

**Fig. 1:**
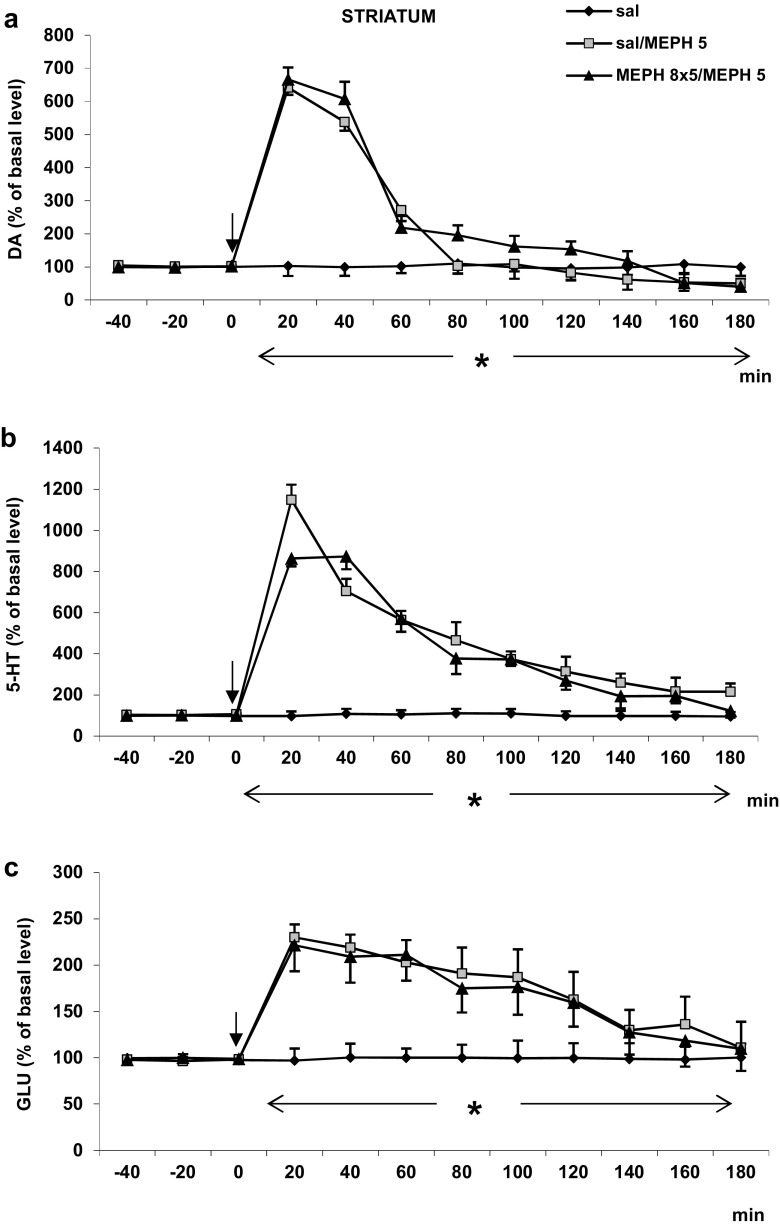
The effect of repeated administration of mephedrone (MEPH, 8 × 5 mg/kg) during adolescence on extracellular level of DA, 5-HT, and glutamate (GLU) measured in adulthood (90 PNDs) in the rat striatum. **a**–**c** The time course. Values are the mean ± SEM (*n* = 4–6 animals per group). Time of drug injection is indicated with an arrow. *P < 0.001, vs. saline/saline group (repeated measures ANOVA and Tukey’s post hoc test)

Mephedrone given repeatedly (8 × 5 mg/kg) during adolescence period significantly increased extracellular DA level in the rat striatum as measured on 90 PNDs in response to the challenge dose of 5 mg/kg (Fig. 1a). The same mephedrone dose also markedly increased extracellular DA level in saline-treated animals (Fig. 1a). Repeated measures ANOVA showed a significant effect of treatment groups [*F*_2,11_ = 114, *P* < 0.0001], sampling period [*F*_8,88_ = 343, *P* < 0.0001], and the interaction between treatment groups and sampling period [*F*_16,88_ = 92, *P* < 0.0002].

The extracellular 5-HT level in the rat striatum was increased to a similar extent by the challenge mephedrone dose of 5 mg/kg both in saline- and mephedrone-treated groups during adolescence period (Fig. 1b). Repeated measures ANOVA showed a significant effect of treatment groups [*F*_2,12_ = 1776, *P* < 0.0001], sampling period [*F*_8,88_ = 653, *P* < 0.0001], and the interaction between treatment groups and sampling period [*F*_16,88_ = 189, *P* < 0.0001].

The extracellular glutamate level was increased to a similar extent by the challenge mephedrone dose of 5 mg/kg both in saline- and mephedrone-treated groups during adolescence period (Fig. 1c). Repeated measures ANOVA showed a significant effect of treatment groups [*F*_2,11_ = 913, *P* < 0.0001], sampling period [*F*_8,88_ = 236, *P* < 0.0001], and the interaction between treatment groups and sampling period [*F*_16,88_ = 62, *P* < 0.0001].

#### Nucleus Accumbens

**Fig. 2:**
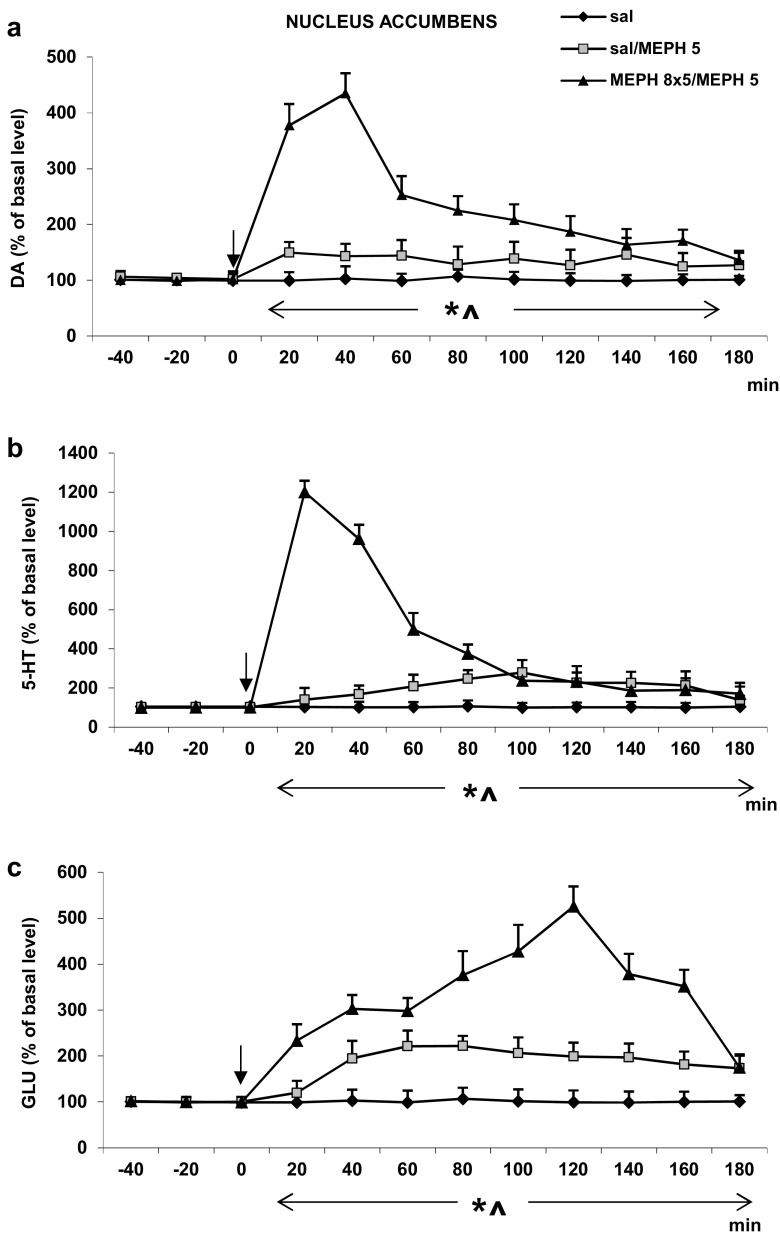
The effect of repeated administration of mephedrone (MEPH, 8 × 5 mg/kg) during adolescence on extracellular level of DA, 5-HT, and glutamate (GLU) measured in adulthood (90 PNDs) in the rat nucleus accumbens. **a**–**c** The time course. Values are the mean ± SEM (*n* = 4–6 animals per group). Time of drug injection is indicated with an arrow. *P < 0.001, vs. saline/saline group; ^*P* < 0.001, vs. saline/MEPH group (repeated measures ANOVA and Tukey’s post hoc test)

The extracellular DA level in response to a challenge dose of mephedrone (5 mg/kg) was lower in saline- than in mephedrone-treated animals during adolescence period (Fig. 2a). Repeated measures ANOVA showed a significant effect of treatment groups [*F*_2,11_ = 604, *P* < 0.0001], sampling period [*F*_8,88_ = 123, *P* < 0.0001], and the interaction between treatment groups and sampling period [*F*_16,88_ = 109, *P* < 0.0001].

The challenge dose of mephedrone (5 mg/kg) increased the extracellular 5-HT level in the rat nucleus accumbens to a lesser extent in saline- than mephedrone-treated animals during adolescence period (Fig. 2b). Repeated measures ANOVA showed a significant effect of treatment groups [*F*_2,11_ = 1342, *P* < 0.0001], sampling period [*F*_8,88_ = 523, *P* < 0.0001], and the interaction between treatment groups and sampling period [*F*_16,88_ = 671, *P* < 0.0001].

The extracellular glutamate level in the nucleus accumbens was more potently increased by a challenge dose of mephedrone (2.5 mg/kg) in mephedrone- than in saline-treated animals during adolescence period (Fig. 2c). Repeated measures ANOVA showed a significant effect of treatment groups [*F*_2,12_ = 242, *P* < 0.0001], sampling period [*F*_8,96_ = 34, *P* < 0.0001], and the interaction between treatment groups and sampling period [*F*_16,96_ = 26, *P* < 0.0001].

#### Frontal Cortex

**Fig. 3:**
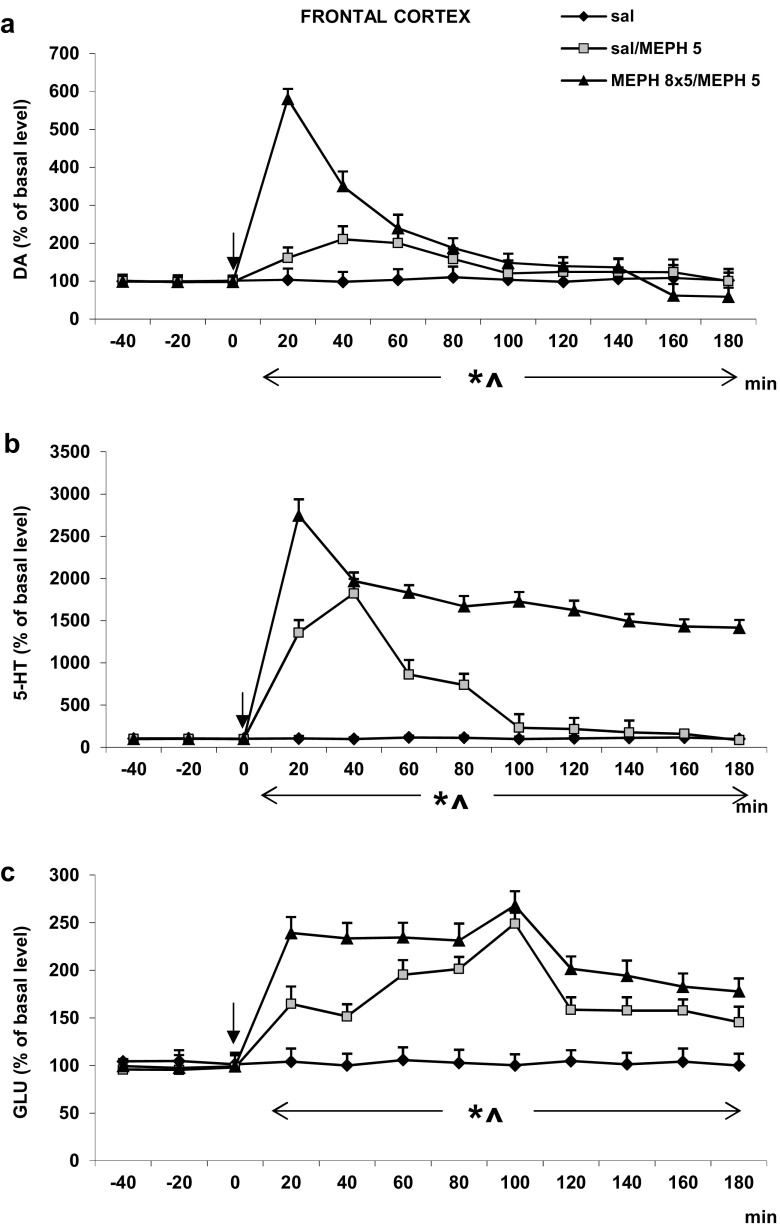
The effect of repeated administration of mephedrone (MEPH, 8 × 5 mg/kg) during adolescence on extracellular level of DA, 5-HT, and glutamate (GLU) measured in adulthood (90 PNDs) in the rat frontal cortex. **a**–**c** The time course. Values are the mean ± SEM (*n* = 4–6 animals per group). Time of drug injection is indicated with an arrow. *P < 0.001, vs. saline/saline group; ^*P* < 0.001, vs. saline/MEPH group (repeated measures ANOVA and Tukey’s post hoc test)

The challenge dose of mephedrone (5 mg/kg) increased extracellular DA level in the rat frontal cortex more potently in mephedrone- than in saline-treated animals (Fig. 3a). Repeated measures ANOVA showed a significant effect of treatment groups [*F*_2,11_ = 309, *P* < 0.0001], sampling period [*F*_8,88_ = 266, *P* < 0.0001], and the interaction between treatment groups and sampling period [*F*_16,88_ = 182, *P* < 0.0001].

The increase in extracellular 5-HT level in the rat frontal cortex induced by the challenge dose of mephedrone (5 mg/kg) was weaker in saline- than mephedrone-treated animals during adolescence period (Fig. 3b). Repeated measures ANOVA showed a significant effect of treatment groups [*F*_2,11_ = 474, *P* < 0.0001], sampling period [*F*_8,88_ = 158, *P* < 0.0001], and the interaction between treatment groups and sampling period [*F*_16,88_ = 61, *P* < 0.0001].

The extracellular glutamate level was increased by the challenge dose of mephedrone (5 mg/kg), but the increase was weaker in saline- than mephedrone-treated animals during adolescence period (Fig. 3c). Repeated measures ANOVA showed a significant effect of treatment groups [*F*_2,11_ = 1030, *P* < 0.0001], sampling period [*F*_8,88_ = 45, *P* < 0.0001], and the interaction between treatment groups and sampling period [*F*_16,88_ = 15, *P* < 0.0001].

### The Effect of Repeated Administration of Mephedrone During Adolescence on the Basal Extracellular Level of DA, 5-HT, and Glutamate Measured in Adulthood (90 PNDs) in the Rat Striatum, Nucleus Accumbens, and Frontal Cortex

The basal extracellular level of DA in the rat striatum and nucleus accumbens at 90 PNDs was significantly (*P* < 0.05) decreased after chronic mephedrone administration during adolescence (Table 1). In contrast, the basal extracellular 5-HT level was significantly (*P* < 0.001) increased in the nucleus accumbens and the frontal cortex (Table 1). Similarly, the basal extracellular level of glutamate was significantly (*P* < 0.001) increased in the rat nucleus accumbens (Table 1).

**Table 1:** Basal levels of DA, 5-HT, and glutamate (GLU) in the rat striatum, nucleus accumbens, and frontal cortex after chronic administration of mephedrone (MEPH, 8 × 5 mg/kg) during the adolescence period and as measured on 90 PNDs

Treatment (mg/kg)	DA (pg/10 μl)	5-HT (pg/10 μl)	GLU (ng/10 μl)
	Mean ± SEM (*n*)		
Striatum			
Saline	18.7 ± 1.6 (5)	0.35 ± 0.06 (5)	0.68 ± 0.11 (5)
MEPH (8 × 5)	10.13 ± 1.34 (5)*	0.38 ± 0.03 (5)	0.48 ± 0.06 (5)
Nucleus accumbens			
Saline	0.79 ± 0.06 (5)	0.12 ± 0.01 (5)	0.48 ± 0.05 (5)
MEPH (8 × 5)	0.43 ± 0.04 (5)*	1.04 ± 0.18 (5)**	1.32 ± 0.16* (5)
Frontal cortex			
Saline	0.57 ± 0.06 (5)	0.14 ± 0.01 (5)	1.02 ± 0.18 (5)
MEPH (8 × 5)	0.71 ± 0.08 (5)	2.49 ± 0.27 (5)**	0.72 ± 0.12 (5)

Data are shown as the mean ± SEM (*n*)

*P < 0.05; **P < 0.001 vs. respective control (one-way ANOVA and Tukey’s post hoc test)

### The Effect of Repeated Administration of Mephedrone During Adolescence on the DA and 5-HT Turnover Rates Measured in Adulthood (90 PNDs) in the Rat Striatum, Nucleus Accumbens, and Frontal Cortex

**Fig. 4:**
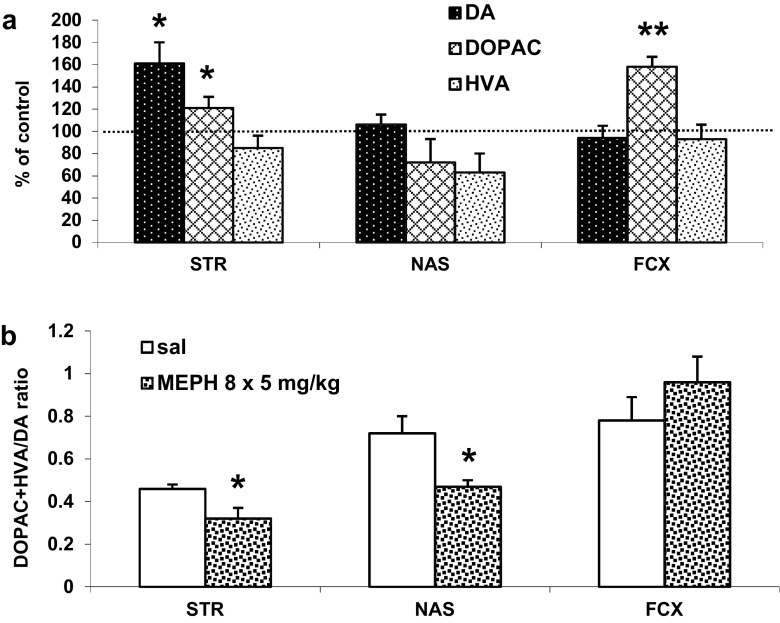
The effect of repeated mephedrone (MEPH, 8 × 5 mg/kg) administration during adolescence on DA turnover rate measured in adulthood (90 PNDs) in the rat striatum (STR), nucleus accumbens (NAS), and frontal cortex (FCX). Data are shown as the mean ± SEM (*n* = 5 animals per group). **a** Percent change in comparison to saline-treated rats and calculated from the absolute numbers given in nanograms/milligram of tissue content presented in supplementary material. **b** (DOPAC + HVA)/DA ratio

**Fig. 5:**
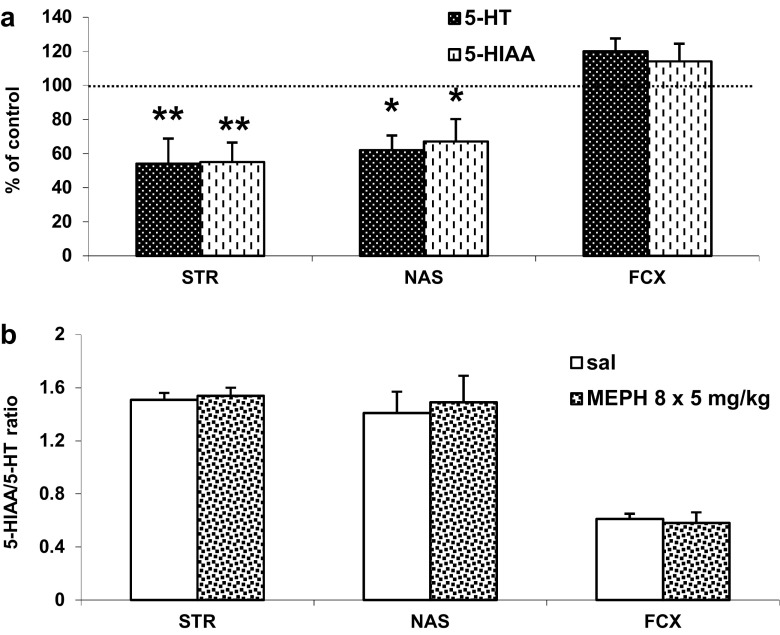
The effect of repeated mephedrone (MEPH, 8 × 5 mg/kg) administration during adolescence on 5-HT turnover rate measured in adulthood (90 PNDs) in the rat striatum (STR), nucleus accumbens (NAS), and frontal cortex (FCX). Data are shown as the mean ± SEM (*n* = 5 animals per group). **a** Percent change in comparison to saline-treated rats and calculated from the absolute numbers given in nanograms/milligram of tissue content presented in supplementary material. **b** 5-HIAA/5-HT ratio

Mephedrone given chronically during adolescence enhanced DA content in the striatum (*P* < 0.05) and DOPAC level in the striatum and frontal cortex (*P* < 0.05 and *P* < 0.001, respectively) to 161, 121, and 158% of control values, respectively (Fig. 4a). The DA turnover rate expressed as (DOPAC + HVA)/DA ratio was significantly decreased in the striatum and nucleus accumbens (*P* < 0. 05 and *P* < 0.01, respectively) and was unchanged in the frontal cortex (Fig. 4b). 5-HT and 5-HIAA tissue level was decreased to ca. 54 and 55% of control level in the striatum and 62 and 67% of control level in the nucleus accumbens (*P* < 0.001 and *P* < 0.05, respectively, Fig. 5a). The 5-HT turnover rate expressed as 5-HIAA/5-HT ratio was not changed in all brain regions studied (Fig. 5b). The absolute values of DA, DOPAC, HVA, and 5-HT and the 5-HIAA levels are given in the attached supplementary material to this paper (Table 2).

### The Effect of Repeated Administration of Mephedrone During Adolescence on Oxidative DNA Damage in the Rat Cortex

**Fig. 6:**
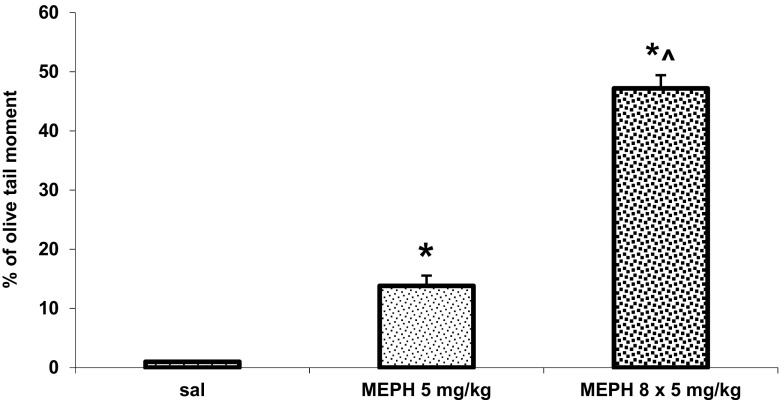
The effect of a single (5 mg/kg) and repeated (8 × 5 mg/kg) administration of mephedrone (MEPH) during adolescence on the oxidative damage of DNA in the nuclei from the rat cortex measured in adulthood (90 PNDs). Data are the mean ± SEM (*n* = 6 animals per group) and represent an olive tail moment shown as the product of the tail length and the fraction of total DNA in the tail. *P < 0.01, in comparison to the control group; ^*P* < 0.01, chronic vs. single administration (one-way ANOVA and Tukey’s post hoc test)

Mephedrone given repeatedly (8 × 5 mg/kg) during adolescence period produced DNA damage shown as a percent of olive tail moment in the rat cortex at 90 PNDs (Fig. 6). The damage was more potent in animals treated chronically with mephedrone than in animals which received a single dose (5 mg/kg) of mephedrone. The lack of difference in DNA damage between the rat whole cortex and the frontal cortex after administration of mephedrone single dose of 5 mg/kg is presented in supplementary material to this paper (Fig. 7).

## Discussion

The findings from this study show that mephedrone exposure during adolescence period facilitates DA, 5-HT, and glutamate outflow in response to the challenge dose, in the frontal cortex and nucleus accumbens but not in the striatum. The decreased tissue content of 5-HT and 5-HIAA in the striatum and nucleus accumbens suggests a possible injury of serotonin nerve terminals. The oxidative damage of cortical DNA indicates a risk of neurotoxic changes in the cortical brain region.

As shown previously by us, mephedrone at a single dose of 5 mg/kg more potently increased 5-HT than DA extracellular level in rat brain regions similar to our present work (Gołembiowska et al. 2016). Other authors studied mephedrone effect in the rat nucleus accumbens. Depending on the dose or route of administration, mephedrone increased extracellular DA and 5-HT levels with a distinct strength. The mephedrone dose of 3.2 mg/kg ip enhanced DA but not 5-HT level to ca. 400% of baseline in Sprague Dawley rats (Suyama et al. 2016). Kehr et al. (2011) showed in the same animal strain that similar mephedrone dose injected sc increased extracellular DA and 5-HT levels to a maximum of 450 and 900% of baseline, respectively. The 10 mg/kg sc dose evoked a greater increase in extracellular 5-HT, compared to DA level (maximum ca. 2000 and 1000% of baseline, respectively) in the nucleus accumbens of Sprague Dawley rats (Wright et al. 2012). Mephedrone (iv, 1–3 mg/kg) elevated DA and 5-HT levels in the nucleus accumbens of Sprague Dawley rats with the magnitude of effect stronger on 5-HT than DA (to ca. 1000–2500 and 300–400% of baseline, respectively) (Baumann et al. 2012; Mayer et al. 2016). Thus, mephedrone is a more preferential releaser of 5-HT than DA which may be a consequence of lower DAT/SERT ratio (Rickli et al. 2015). The differences in potency of the drug effect may be related with the rat strain and the route of administration. The relatively small increase in neurotransmitter levels in the nucleus accumbens after a single ip mephedrone dose observed in our study suggests that Wistar rats are less sensitive to this drug in comparison to Sprague Dawley rats. However, other brain regions, such as the striatum and frontal cortex, were affected markedly with a relatively greater increase in extracellular 5-HT than DA level.

Neurobiological processes occurring in adolescence influence behavior and general skills in adulthood. The use of NPS during this developmental period has been found to be a predictive factor of the drug addiction or mood disorders in adulthood (Chen et al. 2009; Spear 2000). In our study, rechallenge with mephedrone at adulthood after a previous exposure to the drug during adolescence promoted the increased response of DA, 5-HT, and glutamate systems in the rat nucleus accumbens and frontal cortex as compared to saline pretreatment. These data suggest that early exposure to mephedrone may sensitize monoaminergic and glutamatergic neurons to challenge mephedrone dose administered in adulthood and may be a predictive factor of the development of addiction. Surprisingly, the response of striatal DA, 5-HT, and glutamate neurons to the challenge dose of mephedrone was similar in control and mephedrone-pretreated animals which suggests recovery of the striatal circuits from mephedrone treatment in adolescence period. The recovery of 5-HT terminals after repeated administration of MDMA was observed in some regions of non-human primate brains (Scheffel et al. 1998).

Mephedrone administration in adolescent animals produced changes in the basal neurotransmitter levels in all studied brain regions. Accordingly, extracellular 5-HT level was increased in the frontal cortex and nucleus accumbens. The increase in 5-HT was accompanied with enhanced extracellular glutamate level in the nucleus accumbens. The higher extracellular 5-HT level might result from mephedrone-induced disturbance in SERT function in the mesocortical and mesolimbic 5-HT systems. This points to the possibility that the activation of 5-HT2A receptors located on cortical pyramidal cells by endogenous 5-HT may elicit the increased response of DA, 5-HT, and glutamate neurons to the challenge dose of mephedrone in mesocortical and mesolimbic brain regions (Alex and Pehek 2007). This mechanism may be also responsible for the observed increase in the basal glutamate level in the nucleus accumbens but not in the striatum and frontal cortex, where mephedrone did not affect this level.

To assess the potential neurotoxic effect of mephedrone treatment in adolescence period, we studied 5-HT and DA contents as markers of neuronal injury in adulthood. The 5-HT and 5-HIAA deficit (expressed as % of control level) induced by the administration of mephedrone was found in the striatum and nucleus accumbens but not in the frontal cortex. The observed nearly the same decrease in 5-HT and 5-HIAA content in the striatum and nucleus accumbens was not reflected by the turnover rate in these neurons since the 5-HIAA/5-HT ratio was unchanged. These results indicate the possible injury of 5-HT nerve endings and are in agreement with reports of other authors who found the loss of 5-HT nerve terminals after repeated high doses of mephedrone given at elevated ambient temperature to adolescent mice and rats (Hadlock et al. 2011; Martinez-Clemente et al. 2014). On the other hand, the release data did not indicate neurotoxicity to the serotonergic terminals since these terminals seemed to be functional in all studied brain regions. An alternative explanation of this discrepancy between the decreased 5-HT and 5-HIAA tissue content and the increased 5-HT release in response to challenge dose may be based on the weaker stimulation of 5-HT cell bodies in the raphe nuclei by descending glutamatergic pathways from the cortex. Therefore, 5-HT terminals may respond more strongly to the challenge dose to overcome the deficit in neurotransmitter synthesis as shown by our microdialysis experiments. Thus, imbalance in glutamatergic neurotransmission could contribute to adaptive changes in 5-HT cells which receive glutamatergic innervation from cortical regions (Soiza-Reilly and Commons 2011).

Mephedrone pretreatment during adolescence does not appear to cause dopaminergic neurotoxicity in rats as we did not observe DA deficit in adult animals. In contrast, we observed the increase in striatal DA and DOPAC cortical levels and no change in the levels of both compounds in the nucleus accumbens, expressed as percent of respective control groups. The turnover rates expressed as (DOPAC + HVA)/DA ratio indicate inhibition of DA metabolism in dopaminergic nerve terminals in the striatum and nucleus accumbens and no change in the frontal cortex. These results match the data on the low basal extracellular level of DA in the striatum and nucleus accumbens. The lower DA turnover rate and basal extracellular level in the striatum and nucleus accumbens might be caused by a weaker stimulation of postsynaptic pathways projecting to the nigral or ventral tegmental area (VTA) regions (Di Mateo et al. 2008). Thus, mephedrone pretreatment during adolescence period causes long-lasting changes in nigrostriatal and mesolimbic DA pathways but it does not produce injury of DA neurons. Our data are in agreement with results of other authors who did not observe injury of DA nerve endings even after administration into adolescent mice and rats of higher repeated doses of mephedrone than those used in the present study (Hadlock et al. 2011; Motbey et al. 2012; López-Arnau et al. 2015). The lack of damage of DA and 5-HT terminals in the frontal cortex of adult animals might be caused by neuronal recovery. Such lack of neuronal damage as the result of recovery was observed in baboons after administration of MDMA in adolescence period (Scheffel et al. 1998).

It is well known that administration of psychostimulants to rodents results in damage of monoaminergic neurons through the production of reactive oxygen species (ROS) (Cadet and Brannock 1998; Wrona and Dryhurst 2001). Due to similarity of mephedrone to amphetamines, we investigated oxidative DNA damage by ROS with the use of the comet assay. It was shown that mephedrone given repeatedly during adolescence period produced DNA single- and double-strand breaks in the rat cortex of adult animals. The effect of repeated mephedrone administration was stronger than the effect of a single dose of the drug. The means of DNA oxidation by psychostimulants, such as amphetamines or mephedrone, is related to the development of the oxidative stress. Excessive release of 5-HT by mephedrone leads to the formation of highly reactive free radicals, which can damage nuclear DNA (Halliwell and Whiteman 2004) in non-dopaminergic and non-serotonergic cells. Excitatory neurons in the cortex are primarily glutamatergic pyramidal neurons. Subtypes of pyramidal neurons forming excitatory pathways within the cortex also target subcortical structures (Brown and Hestrin 2009). In addition, cortical regions contain inhibitory GABAergic neurons which are forming connections with pyramidal neurons (Gupta et al. 2000). 5-HT release across the forebrain structures can modulate inhibitory and excitatory synaptic activity via 5-HT1A or 5-HT2A receptors, expressed on both excitatory neurons and inhibitory interneurons. The neuromodulatory action of 5-HT depends on the local 5-HT concentration and location of the receptor subtype. The damage of neuronal glutamatergic cell bodies in the cortex projecting to the substantia nigra and VTA may be responsible for the decrease in DA turnover rates in these regions as well as in lower DA basal extracellular levels. The same mechanism may underlie lower 5-HT and 5-HIAA contents in serotonergic neurons projecting from the raphe nuclei to the striatum and nucleus accumbens in animals pretreated with mephedrone in adolescence. The fact that neuronal terminals respond to rechallenge with mephedrone suggests the absence of cellular deaths in studied brain regions. Our findings are in accordance with results of López-Arnau et al. (2015) who found a rise in antioxidant enzymes in the striatum in response to multiple doses of mephedrone. Those authors postulate that striatal tissue is capable of buffering ROS which may explain the absence of dopaminergic injury in this area. In contrast, the frontal cortex was most affected by mephedrone since antioxidant defense was not sufficient in this tissue. In addition, oxidation of polyunsaturated fatty acids was significantly augmented in this brain area (López-Arnau et al. 2015). These data are in accordance with our findings as we observed oxidative damage of DNA in cortical cells in this region. The induction of oxidative stress evidenced by decreased total antioxidant status, increase in malondialdehyde concentration, and increase in catalase activity was also found in the mouse frontal cortex (Budzynska et al. 2015). These data confirm our findings on possible oxidative injury of subpopulation of cortical cells.

Overall, the findings of our study indicate that the administration of repeated low doses of mephedrone during adolescence period affects monoaminergic and glutamatergic neurotransmission, and long-lasting changes in the release of DA, 5-HT, and glutamate in the frontal cortex and nucleus accumbens are apparent in adulthood. Furthermore, mephedrone treatment in adolescent rats does not seem to induce injury of 5-HT and DA neuronal endings. The oxidative stress generated by mephedrone treatment during adolescence period seems to be responsible for the neuronal damage of cortical cells and dysregulation of cortical inputs to subcortical structures and may lead to cognitive deficits that can have long-term consequences. In addition, it may suggest the risk of drug abuse in adulthood.

## Electronic supplementary material

ESM 1 (DOC 23 kb)

ESM 2 (DOC 32 kb)
