# Supplementary material for: The Effects of Exposure to Mephedrone During Adolescence on Brain Neurotransmission and Neurotoxicity in Adult Rats
Source: Neurotox Res. 2018 Apr 30;34(3):525–37. doi: 10.1007/s12640-018-9908-0 (PMC6154178; doi:10.1007/s12640-018-9908-0)
Supplement: Supplementary file 1 — (DOC 23 kb) [file 12640_2018_9908_MOESM1_ESM.doc]

**Fig. 7.** The effect of a single (5 mg/kg) administration of mephedrone (MEPH) during adolescence on the oxidative damage of DNA in the nuclei from the rat frontal cortex and the whole cortex measured in adulthood (90 PND). Data are the mean ± SEM (n = 6 animals per group) and represent an olive tail moment shown as the product of the tail length and the fraction of total DNA in the tail. * *P* < 0.01 in comparison to control group (one-way ANOVA and Tukey’s *post hoc* test).
