# Supplementary material for: The Effects of Exposure to Mephedrone During Adolescence on Brain Neurotransmission and Neurotoxicity in Adult Rats
Source: Neurotox Res. 2018 Apr 30;34(3):525–37. doi: 10.1007/s12640-018-9908-0 (PMC6154178; doi:10.1007/s12640-018-9908-0)
Supplement: Supplementary file 2 — (DOC 32 kb) [file 12640_2018_9908_MOESM2_ESM.doc]

**Table 2.** Tissue contents of DA, DOPAC, HVA, 5-HT and 5-HIAA in the rat striatum, nucleus accumbens and frontal cortex measured on 90 PND after chronic administration of mephedrone (MEPH, 8 x 5mg/kg) during adolescence. Data are shown as the mean ± SEM (n). **P* <0.05, ***P* <0.001 vs. respective control (one way ANOVA and Tukey’s *post hoc* test).

| Treatment  (mg/kg) | DA | DOPAC | HVA | 5-HT | 5-HIAA |
| --- | --- | --- | --- | --- | --- |
| **Striatum** pg/mg wt ± SEM (n) | | | | | |
| Saline | 12118 ±783 (5) | 4012 ± 201 (5) | 1528 ± 150 (5) | 729 ± 50 (5) | 1102 ± 65 (5) |
| MEPH 8x5 | 19450 ± 3636 (5)* | 4848 ± 483 (5)* | 1295 ± 144 (5) | 392 ± 58 (5)** | 602 ± 69 (5)** |
| **Nucleus accumbens** pg/mg wt ± SEM (n) | | | | | |
| Saline | 8536 ± 690 (5) | 3727 ± 307 (5) | 2435 ± 230 (5) | 958 ± 142 (5) | 1237 ± 90 (5) |
| MEPH 8x5 | 9024 ± 851 (5) | 2686 ± 569 (5) | 1546 ± 258 (5) | 595 ± 51(5)* | 833 ± 110 (5)* |
| **Frontal Cortex** pg/mg wt ± SEM (n) | | | | | |
| Saline | 415 ± 46 (5) | 124 ± 10 (5) | 172 ± 27 (5) | 732 ± 60 (5) | 434 ±21 (5) |
| MEPH 8x5 | 390 ± 43 (5) | 196 ± 17 (5)** | 159 ± 20 (5) | 879 ± 65 (5) | 494 ± 52 (5) |
